# Capecitabine-Associated Loss of Fingerprints: A Case Report of a 62-Year-Old Man With Colorectal Cancer Suffering From Capecitabine-Induced Adermatoglyphia

**DOI:** 10.7759/cureus.15519

**Published:** 2021-06-08

**Authors:** Tasneem Dawood, Muhammad Nauman Zahir, Muhammad Afzal, Yasmin Abdul Rashid

**Affiliations:** 1 Medical Oncology, Aga Khan University Hospital, Karachi, PAK; 2 Oncology, Aga Khan University Hospital, Karachi, PAK

**Keywords:** capecitabine, adermatoglyphia, chemoradiation, neoadjuvant, hand-foot syndrome

## Abstract

Background: Capecitabine is a prodrug of 5-fluorouracil (5-FU) and is converted to 5-FU in tumor tissue. Its primary mechanism of action is the suppression of DNA synthesis via inhibition of thymidylate synthetase. It is mostly used for neoadjuvant chemoradiation, adjuvant chemotherapy for colorectal cancer, metastatic breast, and localized and metastatic gastric cancer, among others. Adverse effects of capecitabine include diarrhea, hand-foot syndrome (HFS), pancytopenia, stomatitis, increased bilirubin, nausea, vomiting, and very rarely adermatoglyphia. Dermatoglyphics refers to fingerprints. Adermatoglyphia refers to the loss of fingerprints.

Case review summary: We report the case of a 62-year-old male patient known case of locally advanced colorectal cancer. He presented in the clinic with residual disease after initially being treated with local surgery and chemoradiation with 5-FU. Positron emission tomography (PET) scan done at the time of presentation showed locally advanced disease. He was managed with surgery followed by chemotherapy with oxaliplatin 130 mg/m^2^ and capecitabine (Xeloda) 1500 mg twice a day for two weeks via three weekly cycles. Post cycle five, the patient complained of grade I HFS symptoms and inability to open a bank account due to loss of fingerprints. The patient was oblivious about this condition before that. After completing his adjuvant treatment that is six cycles of oxaliplatin and Xeloda, his symptoms of the HFS and loss of fingerprints, improved.

Conclusion: As this case describes, adermatoglypia is a rare but noticeably side effect of capecitabine with a high chance of reversibility. Similar case reports have been reported with some normalization of fingerprints, after stopping treatment. Fingerprints have been used for centuries as means of identification in banks, aviation, immigration, computers, and mobile phones, amongst others. Awareness regarding the loss of fingerprints due to capecitabine is important for the patient and clinician, and alternative means of identification or other adaptive methods of recognition should be used for these patients.

## Introduction

Capecitabine is an oral pro drug of 5-fluorouracil (5-FU). It is converted to its active form via a three-step activation process with a bioavailability of about 80%. Its mechanism of action involves inhibition of DNA synthesis via inhibition of the enzyme, thymidylate synthetase, needed for DNA synthesis. It is used in the curative and metastatic setting for various cancers. Although it is generally well-tolerated, it does come with side effects. Its adverse effects mainly include diarrhea, mucositis, myelosuppression, paresthesias, hyperbilirubinemia, edema, neuromuscular, and skeletal weakness. Over 50-60% of patients consuming capecitabine develop palmar-plantar erythrodysesthesia (PPE), also known as a hand-foot syndrome (HFS) [1]. It is characterized by redness, swelling, pain, burning, tingling sensation, and tightening of the skin. It might also cause blistering of the skin, mainly the palms and soles of the feet [2]. It is divided into four grades: with higher the grade, the higher the severity of pain and limitation of activity [3]. It is, in some cases, also associated with adermatoglyphia.

Dermatoglyphics refers to the pattern of ridges and grooves on the palms and soles of an individual. Adermatoglyphia refers to the loss of fingerprints [1]. It may be acquired due to medication. In this case report, we will be discussing a male patient with rectal cancer with loss of fingerprints secondary to treatment with capecitabine.

“This article was previously presented as a meeting abstract at the Shaukat Khanum cancer symposium held in Lahore, Pakistan from November 2-4, 2018.”

## Case presentation

A 62-year-old male patient, a retired banker by profession, is a known case of diabetes mellitus, hypertension, locally advanced rectal cancer. He presented to the clinic after being treated earlier at another hospital for rectal cancer. The patient initially had complaints of swelling near the anal region since the last 8-10 months, which was gradual in onset, not associated with pain or per rectal bleeding. He underwent local surgery. His perianal tissue biopsy revealed moderately differentiated adenocarcinoma with cytokeratin 7 patchy positive and CDX 2 as positive. His CT scan of the chest/abdomen/pelvis done in March 2017 revealed diffuse thickening of the anal canal more on its anterior aspect with mild fat stranding in the left ischioanal fossa. Findings were suggestive of a neoplastic lesion along with some post-surgical changes. No regional lymphadenopathy or ascites were seen.

At another hospital, the patient underwent neoadjuvant chemoradiation therapy with 5-fluorouracil till May 2017. The patient was also advised about the surgery but was lost to follow-up secondary to financial constraints. His post-treatment scans done in June 2017 showed thickening in the anal verge, likely suggestive of post-surgical changes. No residual disease seen. In October 2017, he again developed pain in the perianal region along with a foul-smelling discharge. His colonoscopy done in November 2017 revealed rectal growth up to 5 cm from the anal verge. His histopathology revealed moderately differentiated adenocarcinoma. MRI pelvis showed minimal bowel wall thickening involving the anal canal with perilesional edema involving the perianal region, suggestive of post-surgical changes, versus the possibility of residual disease.

He then presented to us, in the clinic in December 2017. His local examination showed ulceration over the anal canal, fixed growth with erythema, and no per rectal bleeding. His positron emission tomography (PET) CT scan was repeated, which showed hypermetabolic circumferential soft tissue mass involving the anal canal, which had progressed. The patient then underwent laparoscopic abdominoperineal resection and local mucocutaneous, fasciocutaneous flap placement on December 23, 2017. Intraoperatively there were no visceral or peritoneal lesions seen. Rectum was completely mobilized beyond the peritoneal reflection. End colostomy was made. Postoperatively patient remained well and made an early recovery. His histopathology specimen revealed moderately differentiated adenocarcinoma, eight lymph nodes were dissected, and lymphovascular and perineural invasion was not present. His pathological stage was ypT3N0. The patient was then started on adjuvant chemotherapy with capecitabine 1000 mg/m^2^ that is 1500 twice daily from days 1-14 and oxaliplatin 130 mg/m^2^ every three weeks.

**Figure 1:**
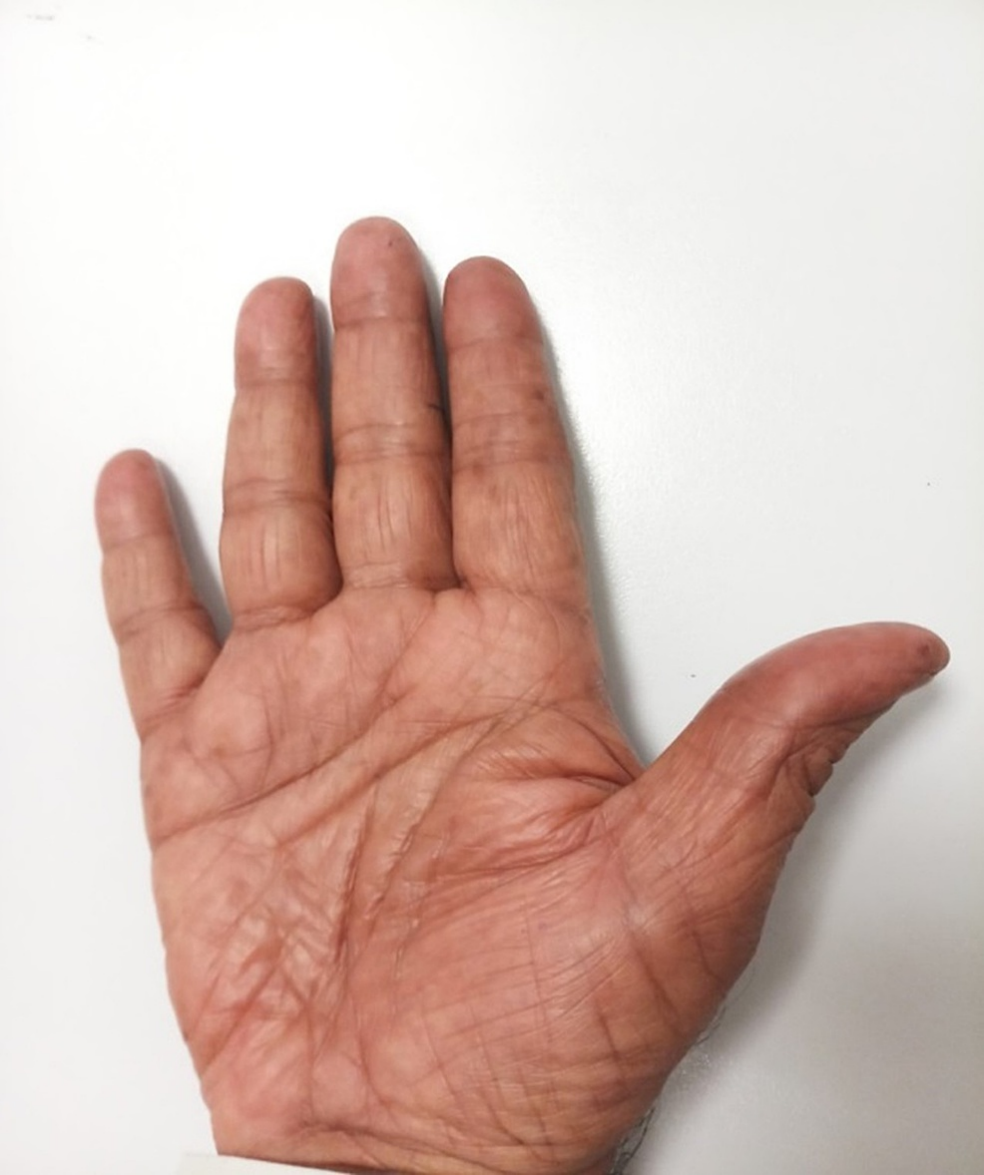
Patient’s loss of fingerprints after five cycles of chemotherapy with capecitabine.

**Figure 2:**
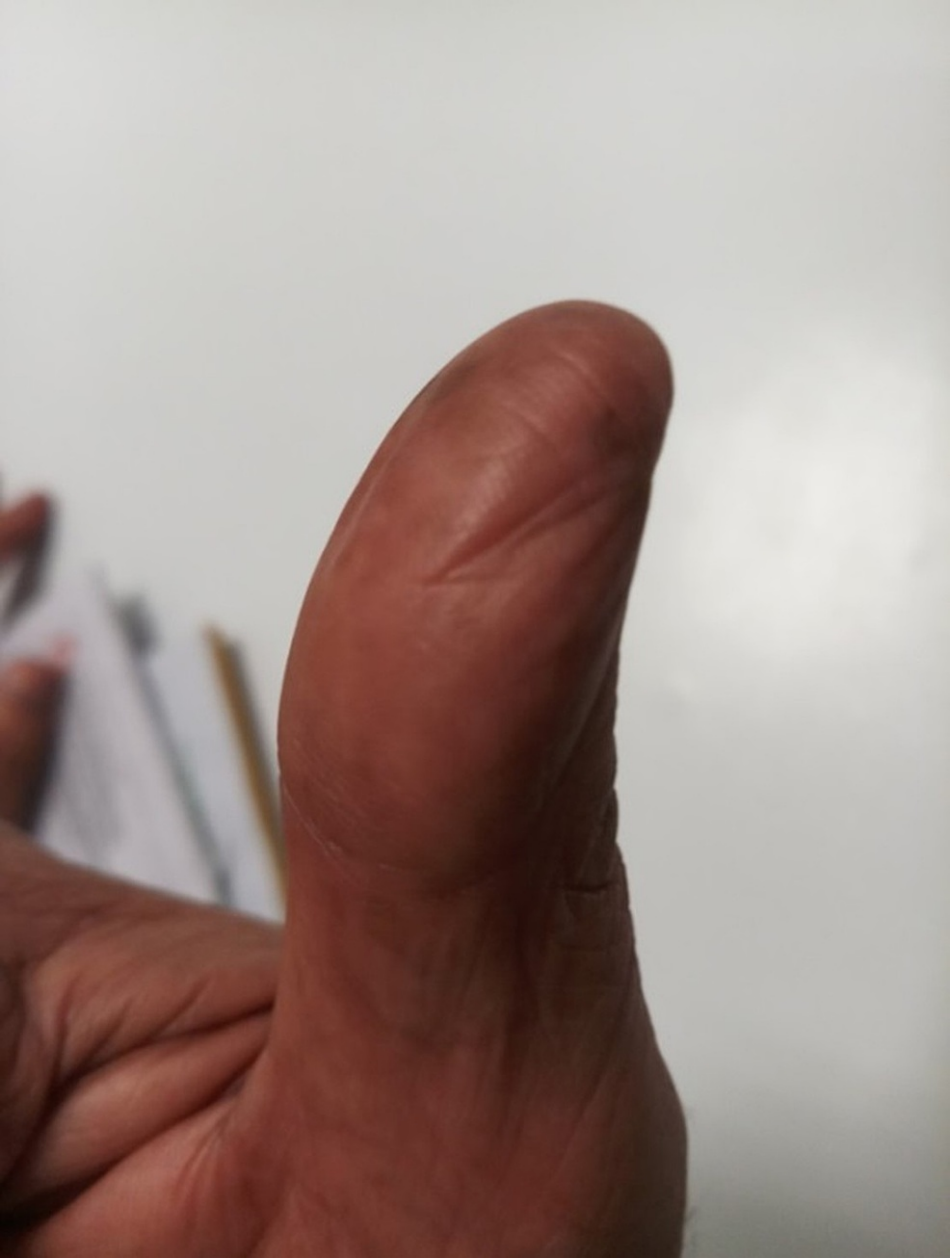
Loss of ridges and grooves of the right thumb.

**Figure 3:**
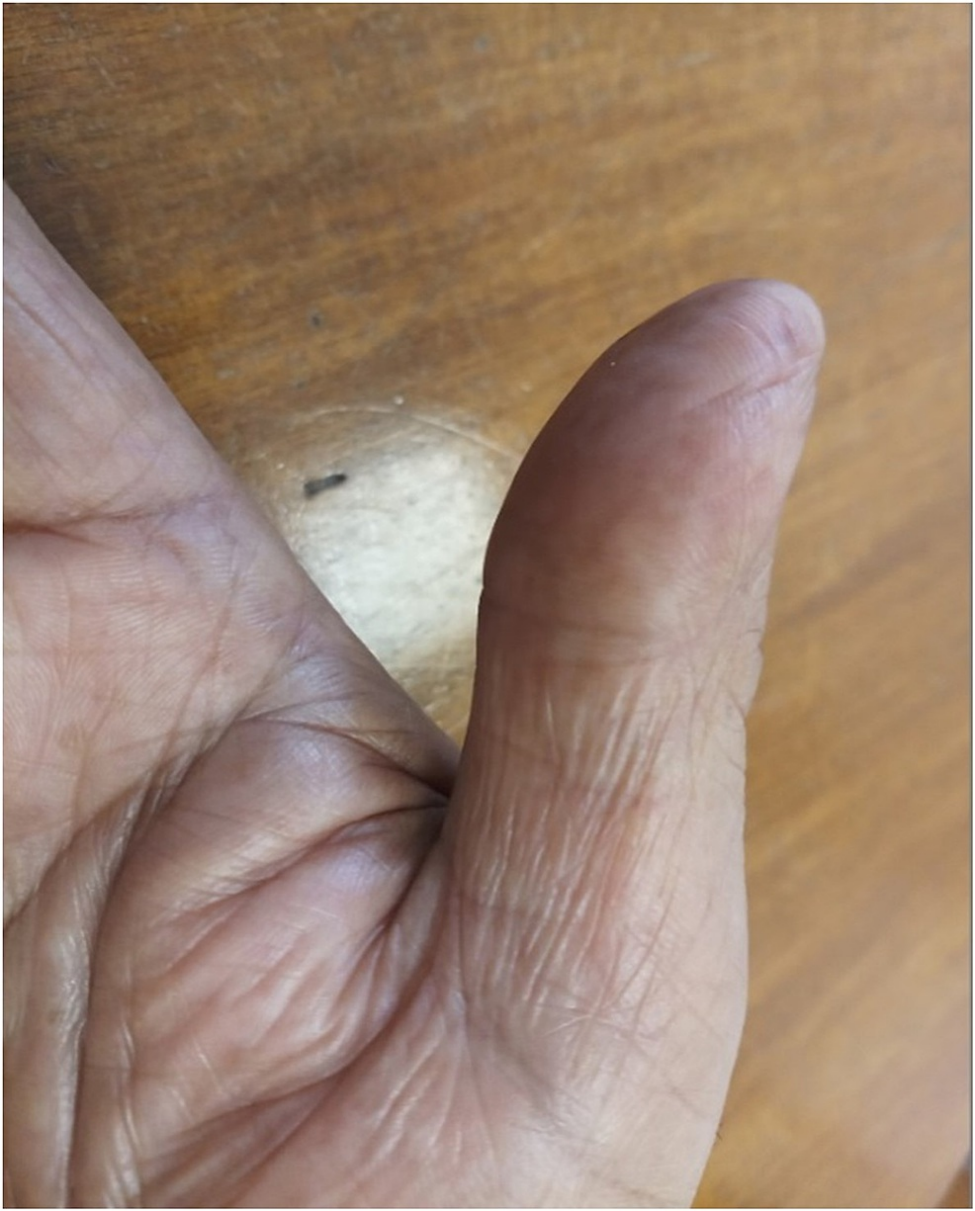
Improvement in fingerprints four weeks after completion of treatment.

**Figure 4:**
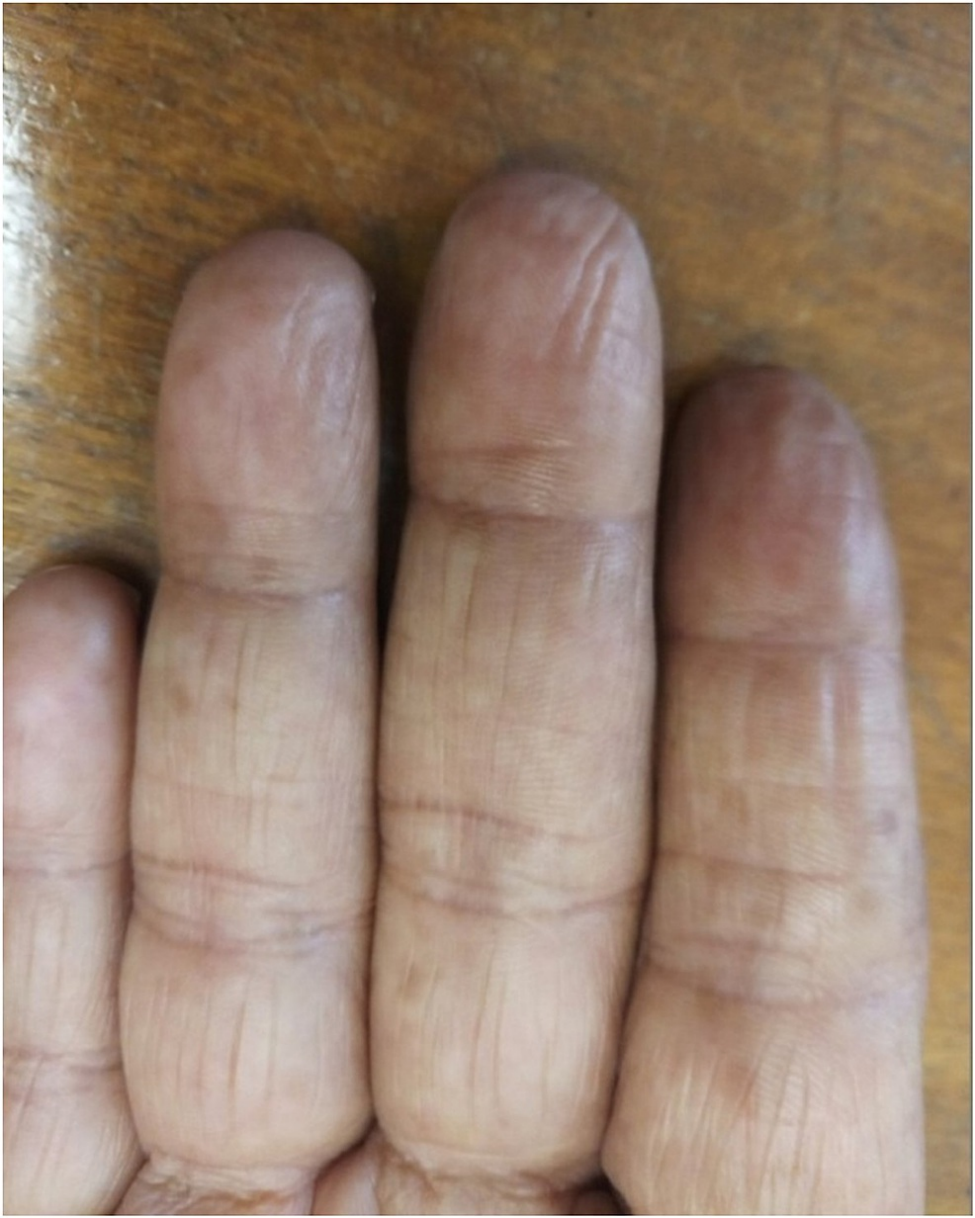
Mild improvement in fingerprints after four weeks of completion of treatment.

After completing five cycles of chemotherapy, he visited the clinic and complained of inability to open a bank account as the patient’s biometrics could not be done since he had lost fingerprints. He had developed hyperpigmentation on the skin of the hands and soles along with numbness in the soles, suggestive of grade I HFS (Figures 1 and 2). The patient also told us about his intention to travel in a couple of months and he might be asked for biometrics at the time of immigration. He completed his sixth cycle in May 2018. His PET CT scan performed after six weeks showed no residual/recurrent disease with a complete metabolic response. By that time his hyperpigmentation had disappeared, and numbness had also improved. His fingerprints had started to appear again visibly after four weeks. His condition had improved without any intervention (Figures 3 and 4).

**Figure 5:**
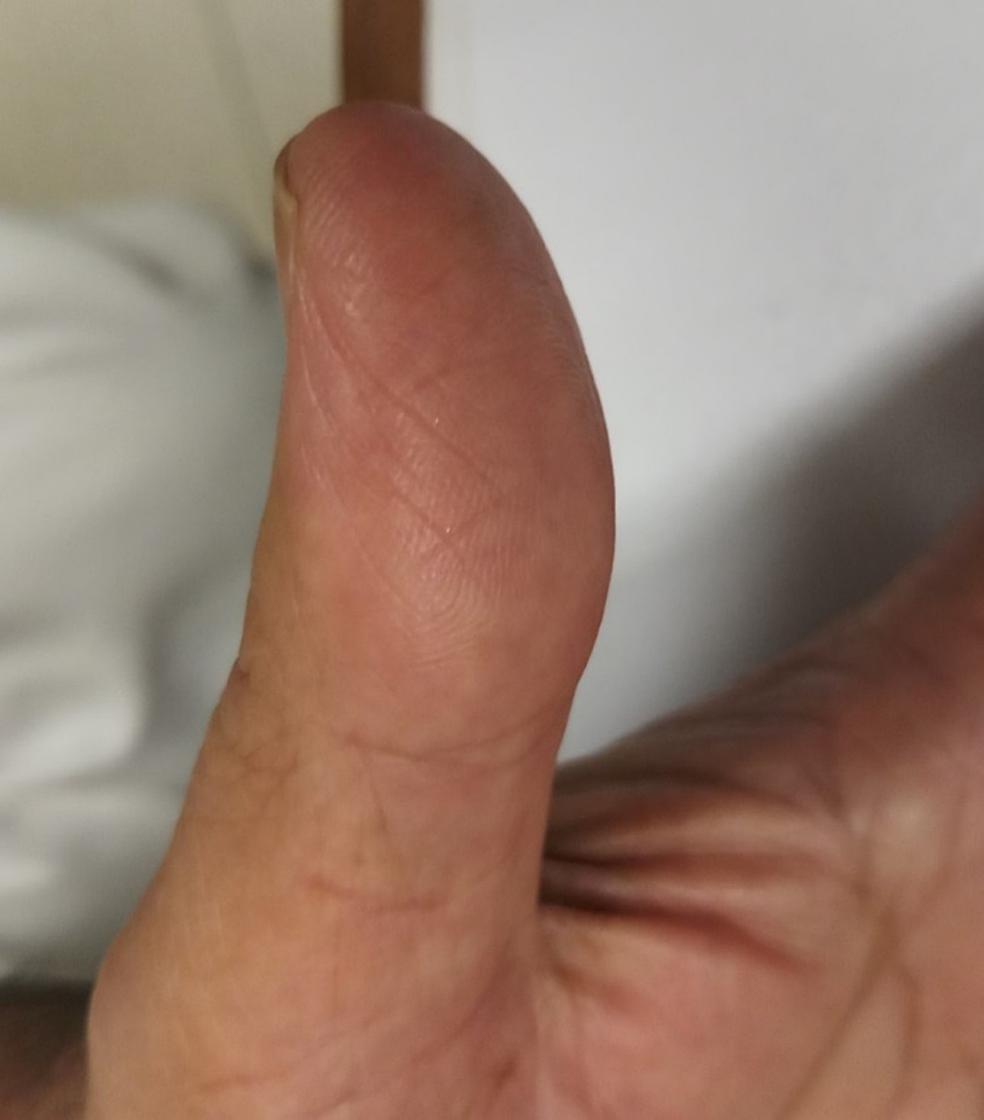
Improvement in fingerprints five months after completion of treatment.

Since then, he has been on surveillance, and he returned to the clinic in October 2018, with improvement in his overall symptoms (Figure 5).

## Discussion

Dermatoglyphics, which refers to the study of fingerprints, comes from Greek Etiology. Fingerprints are an essential tool used for identification, research, criminology, and security. Fingerprints are used as means of identification at the time of immigration, attendance at work, opening a bank account, obtaining a driver’s license, and many such instances [1].

The absence of fingerprints can be inherited or acquired. Inherited forms include adermatoglyphia, also known as immigration delay disease [4]. It is an autosomal dominant disorder caused by a point mutation of the SMARCAD1 helicase gene, resulting in restricted growth of the skin-specific protein [5]. Acquired causes of loss of fingerprints include dermatitis, primary hyperhidrosis, irritant contact dermatitis, atopic dermatitis, dyshidrotic eczema, psoriasis, mechanical abrasion, aging, trauma, burns, and medications [6].

Capecitabine is an oral prodrug of 5-fluorouracil. It undergoes hydrolysis in the liver and tissues to be converted to its active form. Fluorouracil is an antimetabolite that impedes thymidylate synthetase [7]. Fluorouracil appears to be phase-specific for the G1 and S phases of the cell cycle. It is available as a 500 mg tablet, usually taken 30 minutes after a meal. It is primarily used to treat various types of cancers in the curative and metastatic setting, including metastatic breast cancer, triple-negative breast cancer in the adjuvant setting, adjuvant and metastatic colon cancer, neoadjuvant rectal cancer with radiation, and esophageal, gastric cancer, hepatobiliary, pancreatic, neuroendocrine, ovarian, peritoneal cancer, and in many more instances.

The main adverse effects of capecitabine include edema, fatigue, paresthesias, PPE (54-60%), dermatitis, diarrhea, abdominal pain, pancytopenia, hyperbilirubinemia, nausea, vomiting, muscle weakness, skin rash, pigmentation, nail changes, CNS involvement, electrolyte abnormalities, vision changes and many more.

HFS is characterized by an erythematous skin reaction. It was first described in 2003. It involves vascular degeneration of keratinocytes, apoptosis, perivascular lymphocytic filtration, and edema [1]. HFS manifests as dysesthesia, palmar-plantar paresthesias, and erythema at first, and its severity increases to a painful syndrome unless appropriate intervention is done such as topical emollients, pain medications dose reduction, discontinuance in severe cases [8].

The World Health Organization has subdivided HFS into grades depending on its severity and how much it affects daily activities. Grade I is characterized by dysesthesias, paresthesias, with tingling in hand and feet, grade II is swelling with mild pain; grade III has severe pain, ulceration, and erythema with restriction in daily activities. Grade IV is considered as severe desquamation, ulceration, pigmentation, and pain in the palmar-plantar region with severe discomfort in daily activities [8].

No definite cause of HFS has been established. Various hypotheses have been suggested, such as secretion by eccrine glands leads to accumulation of its metabolites, and increased temperature and vascularity can further exacerbate the condition. Another theory proposes that the metabolites cause activation of the cyclooxygenase enzymes promoting inflammation or defective absorption of capecitabine [9]. There are no effective means of preventing or treating the HFS as no proper mechanism of action could be defined. Urea cream can be used as a preventive measure, as some studies have proven its benefit [10]. Nonetheless, this is an area that needs more research and study.

Capecitabine-induced HFS has been observed in 50% of patients treated with it. Capecitabine-associated loss of fingerprints has been reported in patients who have suffered from HFS [1]. The first case was reported in 2009. It was of a 62-year-old man who had metastatic nasopharyngeal cancer, treated with capecitabine for 3 years, and was held at US immigration for his lack of fingerprints. Till 2017, 20 patients have been reported to have a capecitabine-induced loss of fingerprints which has caused them logistic issues such as identification and security concerns [1]. No definite time for the return of fingerprints was mentioned in these cases.

The dose of capecitabine that the patient received in this report was 1000 mg/m^2^, that is 1500 mg twice a day from days 1 to 14 every three weeks for a total of six cycles. No dose reductions were made for this patient as this patient did not have any major side effects. The cases reported earlier have shown that loss of fingerprints was usually associated with the HFS and then even mostly with grades I-II of HFS [1].

Most of the patients in similar scenarios noticed the loss of fingerprints when they had problems with identification at various instances, with variable time durations of loss of fingerprints. However, there is always a possibility that other factors may have played a role in the loss of fingerprints and not just medication use such as the possibility of trauma, eczema, or burns.

## Conclusions

Capecitabine is an oral form of 5-fluorouracil, used to treat various cancers. A common side effect of the medication includes HFS. Another rare, yet noticeable observed side effect includes loss of fingerprints. In this case report, we have discussed one patient who experienced difficulties opening a bank account because of his loss of fingerprints. It is usually associated with low grades of HFS. Most patients are oblivious of the condition until they experience unexpected delays in situations that need biometrics for identification, such as attendance, immigration, and inability to access their phones or security concerns. It is essential to be aware of such instances for both the patient and clinician to be prepared to deal with the circumstances, and arrangements can be made from before such situations. Other adaptive means of identification should also be used be in such instances.
